# What does it mean to follow? A critique of the followership literature and a conceptual model of the emergence of downward following

**DOI:** 10.3389/fpsyg.2023.1072800

**Published:** 2023-09-22

**Authors:** Nicolas Bastardoz, Sofie Adriaensen

**Affiliations:** Department of Work and Organization Studies, Faculty of Economics and Business, KU Leuven, Leuven, Belgium

**Keywords:** follower, downward following, upward leading, process approach, social influence process, shared goals

## Abstract

What does it mean to follow? In this paper, we systematically review the followership literature for the period 2017–2021. Our review shows that the followership literature suffers from three major issues that limit its validity. The followership field is dominated by a role-based approach equating direct reports with followers; empirical studies fail to study actual following behaviors, and there are no studies of downward following, which we define as any behavior or effort aimed at achieving a shared goal, carried out by an individual in a position of formal power who is influenced by one or more individuals in a position of inferior authority. Our manuscript builds on the process approach to study what it means to follow. We argue that the followership field needs to study actual followership behaviors at the micro “interaction episodes” and rely on quantitative behavioral coding. We then propose a conceptual, multi-level model that details antecedents and boundary conditions of the emergence of downward following. We conclude by discussing the organizational implications of our approach and model.

“*Leaders also are followers, and followers also exhibit leadership.*"

Hackman and Wageman (2007, p. 45)

“*The most effective way to be a follower is to know when to lead.*”

Uhl-Bien and Carsten (2018, p. 217)

"*It might sound counterintuitive, but followers do not always follow, any more than leaders always lead.*”

Kellerman (2019, p. 42)

## 1. Introduction

Leadership and followership refer to a social influence process towards the achievement of shared goals (Antonakis and Day, 2018; Yukl and Gardner, 2020). A corollary is that leaders are individuals who have some influence over others in the realization of shared goals and followers are individuals who are influenced and provide efforts towards the realization of a shared goal. To study followers and the followership process, researchers have until now relied on two main paradigms: a *role-based* and a *process* approach (Uhl-Bien et al., 2014; Uhl-Bien and Carsten, 2018). The role-based approach equates followers with individuals in a position of relative low formal power (e.g., Kelley, 1992; Kellerman, 2008; Carsten et al., 2010) whereas the process approach considers followers to be individuals who are influenced to reach shared goals (e.g., Derue and Ashford, 2010; Derue, 2011). Both approaches investigate critical phenomena and are relevant for organizational research.

However, using the same conceptual labels to refer to different phenomena precludes the scientific development of the “followership” field. We argue that the role-based approach, which is generally embraced to operationalize followers, is not in line with the conceptualization of followership as a social influence process. The inconsistency between the conceptualization and operationalization of follower(ship) calls for a fundamental rethink. Contrary to our initial quotes and the role-based approach, we believe it should not be conceptually possible that “followers lead” or that “leaders follow.” As such, the followership field should shift away from its current focus on the role-based approach towards the process approach. Although a role-based approach was appropriate to study following behaviors in traditional, hierarchical organizations with static roles and stable work contexts, a process approach is sorely needed to study followership in flatter organizations with dynamic roles and ever-changing work environments.

The process approach has developed along two different streams. On the one hand, it has relied on a socio-constructionist lens applying discursive, relational, or communicative methods (e.g., Clifton, 2012; Holm and Fairhurst, 2018). These studies are invaluable in generating insights and theories regarding how following emerges. However, they generally focus on leadership emergence, rely on a limited number of cases, and do not test theories. Using a post-positivist tradition, on the other hand, the logic inherent in the process approach has also been applied to the study of emergent (Acton et al., 2019; Badura et al., 2021), shared (D’Innocenzo et al., 2016; Xu et al., 2021), or collective leadership (Yammarino et al., 2012), which investigates how individuals without formal power emerge as leaders or how leadership emerges in informal groups. These studies are equally invaluable to understand the emergence of leaders but have yet offered limited insights into the followership phenomenon (Shamir, 2007; Uhl-Bien and Carsten, 2018), and in particular into how higher-up individuals emerge as followers.

In order to take stock of the followership literature, we first perform a systematic review of followership articles published in top management and applied psychology outlets for the period 2017–2021. Replicating similar findings in the leadership literature (Banks et al., in press; Bedeian and Hunt, 2006; Antonakis et al., 2010; Bastardoz and Day, 2022), our review shows that the followership field (1) equates direct reports with followers; (2) generally neglects the study of actual and dynamic followership behaviors; and (3) has thus far not investigated why, how, and when managers emerge as followers. Building on this systematic review, our manuscript makes three major contributions to the study of followership.

First, *we call for abandoning the generalized practice of equating direct reports with “followers.”* Building on the process approach, we strongly suggest stopping the labeling and assimilation of direct reports as *de facto* followers unless researchers can show that these individuals are (a) influenced (b) to attain shared goals. Our suggestion is in line with the widely-purported and contemporary idea that leaders and followers can emerge from everywhere in an organization (Ashford and Sitkin, 2019). Such a perspective on leadership and followership emergence is theoretically impossible when leading and following are tied to holding a position of formal power in a hierarchy (i.e., role-based approach). In a changing world of work (Kochan and Dyer, 2020) – including more team and work specialization, rapidly changing competitive environments, flatter hierarchies, and a changing workforce – organizations more than ever need that everyone can emerge as a leader and follower.

Second, our review shows that the empirical research on followership using quantitative methods relies almost exclusively on attitudinal, cognitive, or perceptual questionnaires. Unfortunately, questionnaire-based research is not appropriate to study actual and dynamic following behaviors (Antonakis et al., 2010; Sajons, 2020; Hansbrough et al., 2021; Van Knippenberg, 2023) because who follows and leads is inherently dynamic and shifts rapidly (Bastardoz and Day, 2022). We therefore suggest taking an event-based view (Hoffman and Lord, 2013; Morgeson et al., 2015) to focus on leading-following interaction episodes (Katz and Kahn, 1978). More specifically, *we call for more studies using quantitative behavioral coding of natural or in-situ interaction episodes* (Lehmann-Willenbrock and Allen, 2018; Hemshorn de Sanchez et al., 2022), *and in particular studies focusing on actual following behaviors* such as providing efforts towards collectively agreed goals and coordinating one’s actions with other members (Banks et al., in press; Bastardoz and Van Vugt, 2019; Carton, 2022).

Third, we introduce the construct of *downward following*, which we define as *any behavior or effort aimed at achieving a shared goal, carried out by an individual in a position of formal power who is influenced by one or more individuals in a position of inferior authority*. This construct has not yet been identified in the followership literature and is the corollary of upward leadership so that upward leadership and downward followership jointly and simultaneously emerge. Downward following is in line with the process approach and an event-based view because such following behaviors are dynamic and fluid (Oc et al., 2023). For instance, a manager may downward follow during one interaction episode when a direct report uses expertise to lead the workgroup and, only a few moments later, lead the workgroup during another interaction episode when communicating a vision or critical information. Borrowing from various literatures such as voice (Morrison, 2014; McClean et al., 2019), power (Aime et al., 2014; Feenstra et al., 2020) or group processes (Edmondson, 1999), we offer a multilevel conceptual model of downward following using individual predictors and dyadic, task, group, and contextual-level factors as moderators.

## 2. Systematic review

To better grasp the state-of-the-art in followership research, we first conducted a systematic review of the conceptualizations and operationalizations of followership published in top management journals. To do so, we followed best practices for performing systematic reviews (Short, 2009).

### 2.1. Search strategy

Our search focused on the top journals publishing empirical work on leadership and followership. More specifically, we selected journals by impact factor that were listed in the Top 20 of the ‘Management’ category in Web of Science. We eliminated all journals publishing only theoretical or review articles as well as out of scope journals, leaving us with only five journals: *Academy of Management Journal*, *Journal of Applied Psychology*, *Journal of Management*, *Journal of Organizational Behavior*, and *The Leadership Quarterly*. We added the journal *Leadership* because it frequently publishes manuscripts on followership with a more qualitative approach compared to the other five included journals.

We gathered all articles on July 8^th^, 2022, using WebOfScience. We searched for manuscripts including combinations of the word “*follow*” in the text. We focused on articles published between 2017 and 2021 to reflect contemporary practice, resulting in an initial pool of 323 articles. The next step in our search was to include studies that covered any sort of followership constructs (e.g., attitudinal, emotional, cognitive, behavioral, traits). We decided to exclude articles using “followers” as a source to measure leadership perceptions (e.g., a direct report rating a manager leadership style). Both authors manually checked each article for inclusion, and this procedure left us with 137 articles containing 210 distinct followership constructs.^1^

### 2.2. Coding procedure

We decided to focus on eight descriptive variables based on our initial assessment of the followership literature.

#### 2.2.1. Empirical approach

We coded if an article was quantitative, qualitative, or else. The latter category includes non-empirical approaches such as reviews and conceptual articles. Note that if an article used both qualitative and quantitative data, we coded it as quantitative when the data was analyzed statistically. Meta-analyses were coded as quantitative. We only coded for conceptual variables (variables 2 to 4 of our coding) if an article was coded here as “else” or as a meta-analysis; we coded for all variables if an article was coded as quantitative or qualitative.

#### 2.2.2. Construct

This variable lists the followership construct.

#### 2.2.3. Type of construct

We categorized each followership construct in one of the following seven categories: (a) attitudes, (b) emotions, (c) cognitions, (d) behaviors (actual), (e) behaviors (perceptual), (f) behaviors (theory paper); (g) traits or individual differences, and (h) others (including blended or unclear constructs). Note that we differentiated actual and perceptual behaviors based on their operationalization (see variable 8).

#### 2.2.4. Role of construct

We coded for the role played by each followership construct in the conceptual model. Each construct was categorized as (a) an independent variable; (b) a moderator variable; (c) a mediator variable; or (d) a dependent variable.

#### 2.2.5. Approach

We looked at the sample to code for the operationalization of followers. We used the category “role-based approach” when followers were merely direct reports, low-level employees, or individuals labeled as followers without further explanation (e.g., a participant in an experiment interacting with a “leader”). We used the category “process approach” when followers were described as individuals influenced to reach shared goals. The third category, “mixed approach,” contains direct reports or low-level employees for whom there was some indication that they were influenced to reach a shared goal. Finally, we used the category “unclear” when authors did not sufficiently describe what they meant by follower (or leader).

#### 2.2.6. Influence

We coded if the individual labeled as follower had been influenced (broadly defined) or not. When no information was provided regarding any influence between a leader and a follower, and in the presence of a role-based approach, we coded as “no influence.” Examples of “influence” include when followers change their behaviors to be in line with the leader’s vision, the influence of a leader on group decisions, and leadership as a social influence process. If it was unclear from the description whether a “follower” had been influenced, we coded it as “unclear.”

#### 2.2.7. Shared goals

We also coded whether the individual labeled as follower shared some common goals with the leader or not. When no information was provided regarding the presence of shared goals between a leader and a follower, and in the presence of a role-based approach, we coded as “no shared goals.” Examples of “shared goals” include when leaders and followers agree on a goal and extend efforts towards realizing them or when leaders and followers used a collective language. When it was unclear from the authors’ description, we coded it as “unclear.”

#### 2.2.8. Operationalization

We coded for the operationalization of the follower constructs. Examples include scales, experimental manipulation, archival records, economic games, and behavioral coding of videos and records. We also coded for whether the source of the variable was the leader (e.g., leader’s rating of their followers’ OCB) or the follower (e.g., follower’s rating of their trust in the leader).

### 2.3. Results

Table 1, which reports all descriptive statistics from our review, shows that a majority of articles included were quantitative (*N* = 84; 61.3%). The remaining articles were theoretical or review papers (*N* = 48; 35%), and only a few were qualitative (*N* = 5; 3.6%). Attitudinal (*N* = 113 out of 311 coded constructs; 36.3% of all variables), perceptual behaviors (*N* = 69; 22.2%) and cognitive constructs (*N* = 53; 17.0%) represented the majority of followership constructs. Actual following behaviors only made up for a small fraction of all constructs (*N* = 18, 5.8%) along with emotional (*N* = 20, 6.4%), others (*N* = 13, 4.2%), and traits (*N* = 10, 3.2%).

**Table 1:** Summary statistics of systematic review.

Coded variable	Amount	Percent
Empirical approach		
Quantitative	84	61.3%
Others (e.g., theory, review)	48	35%
Qualitative	5	3.6%
Type of construct		
Attitudes	113	36.3%
Behaviors (perceptual)	69	22.2%
Cognition	53	17.0%
Emotions	20	6.4%
Behaviors (actual)	18	5.8%
Behaviors (theory)	15	4.8%
Others	13	4.2%
Traits	10	3.2%
Role of construct		
Dependent variable	153	50.3%
Mediator variable	71	23.4%
Independent variable	47	15.5%
Moderator variable	33	10.9%
Approach		
Role-based approach	76	91.6%
Process approach	0	0%
Combination of role-based and process approach	7	8.4%
Influence		
Individual has not been influenced (or not reported)	76	91.6%
Individual has been influenced	7	8.4%
Shared goals		
Followers have no shared goals with leader (or not reported)	78	94.0%
It is likely that followers have shared goals with leader	5	6.0%
Operationalization (Source of measurement)		
Follower	163	82.3%
Leader	35	17.7%
Measurement		
Scale	166	83.8%
Experimental manipulation	12	6.1%
Qualitative approaches	12	6.1%
Behavioral coding	4	2%
Other	4	2%

Followership constructs mainly served as outcome-based constructs such as dependent (*N* = 153 out of 304 coded constructs, 50.3%) or mediator (*N* = 71, 23.4%) variables. In about one-fourth of the coded constructs, they served as explanatory variables, either as an independent (*N* = 47, 15.5%) or as a moderator (*N* = 33, 10.9%) variable. Our review indicates that a vast majority of followership variables were measured using scales (N = 166 out of 198; 83.8%), and the remaining measurements included (among others) experimental manipulations, case analysis, archival records, and behavioral coding. The data source for followership variables is mostly “followers” themselves (N = 163 out of 198 variables; 82.3%) with only a minority being their “leaders” (N = 35; 17.7%).

All “followers” in our reviewed studies referred to direct reports, individuals in low power positions, or individuals labelled as “followers” (i.e., role-based approach). That is, no studies explicitly investigated the emergence of followers from a process approach. Our review identified seven articles (Meinecke et al., 2017; Molenberghs et al., 2017; Stam et al., 2018; Boulu-Reshef et al., 2020; Gilani et al., 2020; Güntner et al., 2020; Van De Mieroop, 2020) that investigated followers who were partly operationalized as representing influenced individuals (i.e., a combination of role-based and process approaches). However, in all seven cases, followers were individuals who were already considered to be “follower” before the study took place and so did not emerge as followers during the social interaction under study. Out of these seven articles, only five included followers as having some sort of common or shared goals with the leader (Meinecke et al., 2017; Molenberghs et al., 2017; Boulu-Reshef et al., 2020; Gilani et al., 2020; Van De Mieroop, 2020).

## 3. Review discussion

Our systematic review uncovered three critical issues regarding the followership literature. First, *research on followership is dominated by the role-based approach, which operationalizes followers as individuals in low position of power or authority*. In other words, the study of followers is the study of direct reports. The followership field does not actually consider whether a “follower” (a) is influenced or (b) shares some common goals with a “leader.” Second, *the research on followership does not study actual, dynamic behaviors*. Instead, the followership field investigates followers’ attitudes, cognitions, or perceptions of behaviors measured primarily with scales. The field would likely benefit from a re-balance towards actual and dynamic behaviors. Third, *our review documents that downward following is currently unexplored*. We ponder whether this phenomenon is non-existent, uninteresting, or simply not important for organizations. We now discuss each of these three critical issues in more detail and offer concrete solutions to help redirect the field.

### 3.1. Issue #1: direct reports are *de facto* followers

Our review unequivocally shows that the role-based approach, which equates followers with direct reports or subordinates, dominates the study of followership (91.6% of the reviewed papers). The role-based approach is represented in mainstream followership streams such as implicit followership theories (Sy, 2010), followership role orientations (Carsten et al., 2010) and typologies of followership (Kelley, 1992, 2008; Kellerman, 2008; Chaleff, 2009). Carsten (2017) summarized this rank-based view, arguing that the field of “followership considers the skills, behaviors, and influence that individuals use while interacting with “higher-ups” in an effort to advance the mission of the organization” (p. 144). By extension, this approach considers that leaders are individuals in position of authority or power such as managers, supervisors, or bosses.^2^

However, many scholars in the leadership and followership literatures have criticized the essence of the role-based approach to study followership and leadership (e.g., Bedeian and Hunt, 2006; Stech, 2008; Derue, 2011; Ashford and Sitkin, 2019; Larsson and Nielsen, 2021; Bastardoz and Day, 2022). Interestingly, almost a century ago, Cowley (1928) already differentiated between a leader (“an individual who is moving in a particular direction and who succeeds in inducing others to follow after him,” p. 145) and a head[wo]man (“an individual who, because of ability or prestige, has attained to a position of headship,” p. 146). Unfortunately, the fields of leadership and followership have never seriously embraced this call to distinguish leaders from individuals having formal power. Instead, the role-based approach is ubiquitous and is further reinforced by mainstream representations of leaders as CEOs, country Presidents, or individuals with large communities of “followers” on social media.

A role-based approach conflicts with the view that leadership and followership consist in an influential process, and that an individual must influence another to emerge as a leader. Individuals in a low position of formal power are not *de facto* “followers” if they are not influenced (Bedeian and Hunt, 2006) or do not agree on shared goals (Bastardoz and Day, 2022). This misalignment between the conceptualization (i.e., who we say we study) and operationalization (i.e., who we actually study) of followers impedes the scientific progress of the field and limits its validity. Interestingly, a role-based operationalization is also inconsistent with dictionary definitions that consider followers as individuals “in the service of one another,” “that follows the opinions or teaching of another,” or “that imitates another” (https://www.merriam-webster.com/dictionary/follower).^3^

#### 3.1.1. Solutions to issue #1: consider followers as individuals who are influenced to reach goals shared with a leader

To reduce the gap between the conceptualization and operationalization of followers, we argue that the followership field should embrace a “process” approach and consider followers to be individuals who are influenced to reach a shared or common goal. Based on the current state of the field, we call for a moratorium of studies treating direct reports as *de facto* “followers.” We do not aim to imply that studying direct reports or subordinates is irrelevant, to the contrary, but the norm of labeling direct reports as followers is incorrect and creates conceptual confusion. Therefore, researchers using the role-based approach should use another terminology than “follower” to label direct reports, subordinates, collaborators, or employees. Even if that shift may reduce the size of the followership field, it is conceptually correct and an alluring opportunity to re-build on more precise theoretical bases.

Our suggestion to embrace the process approach builds on the “adaptive leadership” framework (Derue and Ashford, 2010; Derue, 2011) and focuses on influence and deference towards shared goals as indicators of leadership and followership. More specifically, it considers that followership is (a) a dynamic state; (b) contextually embedded; and (c) an outcome of a specific influential process.

A process view considers followership as a dynamic and fluid state rather than a static role with fixed labels and positions (Stech, 2008; Derue and Ashford, 2010; Derue, 2011; Sy and Mccoy, 2014). Individuals can instantaneously switch roles that best suit the group or organization’s needs (Sy and Mccoy, 2014). For instance, a team may have a formal manager being accountable for the group outcomes, but this person may in certain situations switch roles by following other group members who have more knowledge, expertise, or information (French and Raven, 1959; Raven, 1993). As the task evolves, different individuals may emerge as followers so that following behaviors shift across group members who have the least knowledge, expertise, or information required. Thus, who emerges as follower varies across the requirement of situations and contexts (Bastardoz and Van Vugt, 2019).

If followership implies being influenced to reach shared goals, it also implies that followership is a result or an outcome of a social process (see Drath et al., 2008 for a similar argument regarding leading and leadership). More specifically, individuals emerge as followers when they (a) are influenced (Shamir, 2007) to (b) reach a common or shared goal (Baird and Benson, 2022). Contrary to good followers in the role-based approach who have independent thoughts and are encouraged to think by themselves (e.g., see Kelley, 2008), good followers in the process approach are individuals willing to be influenced and set aside their own personal goals to pursue a shared goal (see also Bastardoz and Van Vugt, 2019). Seen from this angle, *the study of followership aims to understand why, how, when, and for how long individuals are influenced to reach a shared goal*.

We also disagree with authors who imply that the term “follower” can apply to both direct reports and influenced individuals working on shared goals (e.g., Uhl-Bien and Carsten, 2018). For instance, Carton (2022) considers leaders as individuals who attained a certain position (akin to a role-based approach) and leading as the enactment of an influential process (akin to a process approach). We contend that these two approaches cannot use a similar terminology, at the risk of creating confusion. Leaders and leading, and *de facto* followers and following, cannot be constructs that are so fundamentally disconnected. How can it be possible that “leaders do not lead,” or “followers do not follow”? We argue that it should not be theoretically possible. For theoretical clarity and in line with conceptualizations of leadership and followership, leaders must refer to individuals who temporally influence others towards the achievement of shared goals (i.e., lead), and followers must refer to individuals who are temporally influenced towards the achievement of shared goals (i.e., follow).

### 3.2. Issue #2: lack of focus on actual and dynamic followership behaviors

Our systematic review revealed a second major issue related to how the concept of followership is operationalized. Specifically, most of the variables focus on attitudes, cognitions, or perceptions of behaviors, rather than directly assessing behaviors themselves (see also a recent review of the followership field with similar results by Oc et al., 2023). This is an issue because a broader number of constructs refer to actual behaviors. This finding replicates what Banks et al. (in press) found in the leadership literature, and echoes (Baumeister et al., 2007) criticism of the psychology literature. This situation is inappropriate and threatens the validity of followership research because of three main reasons.

First, variables that are measured with scales such as attitudes, cognitions, and perceptions or evaluations of behaviors (79% of the variables studied in our review) are endogenous and will lead to biased and inconsistent estimates when treated as antecedents in an empirical model (Sajons, 2020; Bastardoz et al., 2023). Given the nature of the human cognition to fill blanks, categorize individuals, and rely on salient perceptual memories (Lord et al., 1978, 1984; Cronshaw and Lord, 1987), idiosyncratic unmeasured factors affect attitudes, cognitions, and perceptions. For example, perceptions of actual behaviors will be biased because each individual has their own sensemaking, implicit theories (i.e., prototype matching), and inferential process that filter their recollections of memory and events (Lord et al., 1978). Furthermore, omitted variables, reverse causality, and simultaneity frequently affect scale measures and thus introduce endogeneity and threats to causal inference (Antonakis et al., 2010, 2016; Wulff et al., 2023). As a case in point, Güntner et al. (2020) show how a subordinate’s behavior affects and is simultaneously affected by a management style, rendering any scale measures of these constructs endogenous and causally invalid. Furthermore, scales can hardly account for the dynamic and flexible nature of followership suggested by the process approach.

Second, the few concrete and actual behaviors uncovered in our review mainly concern behaviors of direct reports that do not fit with a process view of followership. We found some behaviors that do not refer to being influenced such as prohibitive or promotive voice (Kim and Toh, 2019), absenteeism (Nevicka et al., 2018) and free-riding (Boulu-Reshef et al., 2020). Some behaviors also focus on individual goals that inherently differ from shared goals such as perpetrated rudeness (Kluemper et al., 2019), resistance (Güntner et al., 2020), dissent (Uhl-Bien et al., 2014), proactive behaviors (Fuller et al., 2015), and independence (Carsten, 2017). All these behaviors are important and must be studied in their own right (e.g., see the large and blooming literature on *employee* voice; Morrison, 2014, 2023);^4^ however, these behaviors do not pertain to the followership field because they do not refer to individuals being influenced to reach a shared goal.

Third, major taxonomies of followership behaviors (or styles) (Kelley, 1992, 2008; Kellerman, 2008; Chaleff, 2009), which are in line with a role-based approach because they imply that employees’ behaviors are static and context-free, remain influential in the field. These taxonomies provide critical insights about subordinates’ effectiveness, engagement, and courage. However, these taxonomies are limited because they categorize all individuals in a very limited number of types (four in Chaleff, five in Kelley and Kellerman). Also, direct reports’ behaviors result from a myriad of factors (e.g., the direct report’s traits and motivation but also the supervisor’s characteristics and the organizational context; see Brown and Thornborrow, 1996) rendering them endogenous (Bastardoz and Van Vugt, 2019; Güntner et al., 2020). These taxonomies imply that managers have limited responsibility if they face bystander, alienated, or passive employees because it is the nature of individuals to behave as such; however, such reasoning is probably misleading because a majority of individuals become bystander, alienated, or passive in the presence of situations or individuals rendering them as such (e.g., a bad manager). Overall, we encourage followership researchers to refer more cautiously to these taxonomies.

This section indicates that the followership field mainly fails to study actual followership behaviors. We now discuss a way forward to account for following behaviors in line with a process approach.

#### 3.2.1. Solution to issue #2: study actual following behaviors at the micro-interaction episode using quantitative behavioral coding

Along recent calls to study joint (Van Knippenberg and Dwertmann, 2022) and dynamic (McClean et al., 2019) leader and follower behaviors, we need more studies that (a) focus on actual following behaviors; (b) investigate dynamic follower emergence through micro “interaction episodes”; and (c) rely on quantitative behavioral coding.

##### 3.2.1.1. Actual followership behaviors

In our review, we uncovered interesting studies focusing on actual behaviors such as followers’ effort to implement a project (Sloof and Von Siemens, 2021), followers’ willingness to cooperate with a leader (Van de Calseyde et al., 2021), and level of cooperation (Ahmad and Loch, 2020). Other conceptual and review articles also offer concrete behaviors such as the accomplishment of objectives (Behrendt et al., 2017) and loyalty (Ciulla, 2020). Still, the field needs more studies investigating the kind of following behaviors individuals take when granting influence and/or accepting someone else’s goal. We argue that it would be beneficial to focus on proxies of social influence such as individuals’ cooperation and coordination of their activities (Bastardoz and Van Vugt, 2019) or supporting initiatives (Baird and Benson, 2022).

We must clarify two aspects for theoretical precision. First, it is critical to distinguish between proximal and distal followership behaviors. Proximal followership behaviors are short-term, quickly activated, and include being influenced to set one’s personal interest aside (i.e., cooperate) and engage in efforts to realize shared goals (i.e., collaborate). Distal followership behaviors are more long-term and emerge as a result of proximal behaviors. These behaviors include for instance task or group performance, sustained effort, and creative solutions. Second, following behaviors must relate to behaviors that an individual would not have engaged in otherwise. If individuals engage in actions – such as producing efforts or collaborating with others – that they would have anyway performed (in a hypothetical counterfactual state without the influence of a “leader”), there are no following behaviors because no influence happened. *To showcase the emergence of followers, one must show that influence happened or that a shared goal prevailed over a private goal*.

##### 3.2.1.2. Micro-interaction episodes of followership emergence

Because the majority of studies in our review measured followership constructs using retrospective scales, we encourage studies of followership to take an event-based view (Hoffman and Lord, 2013; Morgeson et al., 2015) and focus on micro-interaction episodes, which allows for the study of behaviors in a dynamic and contextual way (Bastardoz and Day, 2022). Building on Katz and Kahn (1978) “role episodes” (pp. 194–195), an interaction episode refers to a social encounter between two or more individuals (e.g., dyadic meetings, group sessions, townhall meetings) whereby individuals act in line with role expectations. These episodes allow studying followers’ emergence through granted influence or acts of followership (similar to “acts of leadership” in Ashford and Sitkin, 2019, p. 458).

Such a lens can help identify how, why, and when different individuals switch between roles across interaction episodes. As an example, Aime et al. (2014) found that teams in which members dynamically follow when situationally required (i.e., power heterarchy) were more creative. Identifying why or how a direct report, a manager, or an executive follows can offer critical insights to predict the effectiveness of groups and organizations. Similarly, it is critical to identify the leading-following patterns across repeated interactions of effective groups and organizations, and how these patterns are affected by environmental and situational constraints. Although calls have been made to study teamwork (Mortensen and Haas, 2018) or leadership (Ashford and Sitkin, 2019; Bastardoz and Day, 2022) from an interaction episode lens, a recent review of the behavioral research on leader-follower interactions suggests that very few studies account for the interconnected patterns of behaviors between group members (Hemshorn de Sanchez et al., 2022).

##### 3.2.1.3. Quantitative behavioral coding

A focus on interaction episodes and *in situ* practices to study followership is currently the province of socio-constructionist or interpretivist epistemological lenses (which may explain why the “process” approach had originally been labeled the “constructionist approach” in Uhl-Bien et al., 2014). For instance, qualitative research on leadership and followership has focused on interaction episodes using observational studies (e.g., Klein et al., 2006; Metiu and Rothbard, 2013), video recordings (Van De Mieroop et al., 2020; Barfod et al., 2022), interviews (e.g., Benson et al., 2016) or case studies (Blom and Alvesson, 2014). This field of research is rapidly expanding to studying situated identity construction (Clifton, 2017), the risks associated with embracing a followership identity (Larsson and Nielsen, 2021), and how the use of authoring claims and grants interacts with and co-creates notions of shared and hierarchical leadership (Holm and Fairhurst, 2018). Such empirical work is necessary to increase our understanding and generate new theories.

The field of followership should complement these studies by engaging in quantitative behavioral coding of natural or in situ interaction episodes. Observational studies or video recordings that focus on the occurrence of certain proximal followership behaviors (e.g., granted influence, levels of effort, cooperation on group tasks) are particularly well suited for quantitative behavioral coding. Such behavioral coding can then be analyzed quantitatively using methodologies such as lag sequential analysis (Güntner et al., 2020) or pattern analysis to uncover temporal and causal patterns (see Lehmann-Willenbrock and Allen, 2018 for an overview). Quantitative behavioral coding is slowly emerging in the leadership literature although it still remains sparsely used (Hemshorn de Sanchez et al., 2022). For instance, Gerpott et al. (2019) coded for verbal leadership behaviors of team members (i.e., task-, relationship-, or change-oriented) and found that specific patterns of behaviors at different times of a project life predict leadership emergence. Similarly, Lee and Farh (2019) investigated the temporal effect of constructive and supportive contributions on leadership emergence, and found that constructive contributions related more strongly to leadership emergence early (i.e., in the idea generation phase) rather than late in the project lifecycle (i.e., in the idea enactment phase). Both articles highlight the importance of a contextual and dynamic view to leading and following.

In summary, we are convinced that there is a large untapped potential to study actual following behaviors at the interaction episode using quantitative behavioral coding. Such micro-level studies will offer a more fine-grained, dynamic, and situationally-embedded understanding of followership.

### 3.3. Issue #3: we have no studies of downward following

Our review highlights a lack of studies investing the emergence of followership from individuals in positions of power. Due to its overreliance on the role-based approach, the field focuses almost exclusively on formal followership, that is, when a direct report follows a supervisor.^5^ However, formal followership and top-down leadership are not always desirable or required. Formal followership works relatively well in presence of a simple task or routine with clear performance standards and stable conditions (Lee and Edmondson, 2017). In such strong situations (Meyer et al., 2010), downward following offers little advantage. For instance, Wellman et al. (2020) found that an inverse pyramid-shaped hierarchy increases organizational performance when task variety is high (but not when task variety is low). When the task is complex and requires decentralized expertise, skills, or information, organizations may benefit from managers who follow their direct reports.

The idea that everyone – and particularly individuals higher-up in organizations – can emerge as a follower is not inherently new. Currently, whenever a process approach is applied to the study of leaders and followers, it generally zooms in on leadership constructs such as leader emergence or shared leadership (Acton et al., 2019; Hanna et al., 2021; Xu et al., 2021). Although many calls have been made to study informal leader emergence (Badura et al., 2021), non-designated leaders (Ashford and Sitkin, 2019) or pure leadership (Bastardoz and Day, 2022), few calls have been made to study its followership counterpart, that is, downward following.

#### 3.3.1 Solution to issue #3: study downward following

Downward following refers to *any behavior or effort aimed at achieving a shared goal, carried out by an individual in a position of formal power who is influenced by one or more individuals in a position of inferior authority*. Downward following necessarily emerges in presence of upward leading, which represents behaviors enacted by one or more individuals with a lower position of authority that influences an individual in a position of formal power towards the realization of shared goals. However, the downward following construct is currently missing in the following literature.^6^ Why is that the case? Is it because downward following does not exist or is simply a rare phenomenon? Is it because downward following is not important or relevant for organizations? We would reject these explanations because downward following is not only common in many (though not all) organizations, but also critical to their effectiveness. One reason for the dearth of studies on downward following behaviors may be that such phenomenon is hard to study with standard quantitative methods. Moreover, researchers using these methods (us included!) are not trained to study a construct inherently dynamic and volatile. Another possible reason may be that we lack a conceptual framework and nomological network to investigate the emergence of this phenomenon, which we aim to provide below (see section 4).

In a changing world of work (Kochan and Dyer, 2020) characterized by (a) more knowledge work and complex tasks, (b) uncertain and rapidly changing business environments, and (c) virtual, self-managed, dispersed, agile, or fluid teams, it becomes quintessential to organizations to create cultures and climates that allow for fluid and dynamic role switching between leading and following. For instance, because teams are no longer located in the same space or have a stable membership (Mortensen and Haas, 2018), it creates various and specific needs for leading and following behaviors that cannot all be resolved from one top-down leader. Indeed, the nature of problems faced today by teams and organizations is becoming so complex and changes are so rapid that more people are required to help solve these problems, suggesting a pressing need for all sorts of following behaviors, including downward following.^7^

We submit that organizations encouraging and promoting an “everyone should follow” mindset will over the long term perform better than organizations focused on hierarchies and formal followership. In a different business environment than today, Katz and Kahn (1978) already noted that “organizations in which influential acts are widely shared are most effective” (p. 332). Extensive empirical evidence supports this claim by showing that teams or organizations with distributed acts of leading and following perform better and are more creative (Taggar et al., 1999; Erez et al., 2002; Aime et al., 2014; Derue et al., 2015; Ziegert and Dust, 2021).

Downward following is instrumental for organizations because it has motivational capabilities. Organizations face a war for talent and a certain disengagement of their workforce (as suggested by the recent “great resignation” and “quiet quitting” discussions), and thus must continuously strive to engage their workforce and maintain their morale up. Self-determination theory (Ryan and Deci, 2000; Van den Broeck et al., 2016) and work design theory (Parker et al., 2017) suggest that employees will be motivated to perform their tasks well when they receive opportunities to grow or master their competence. Downward following and upward leading thus become a strategy to engage employees who are willing to have an impact and search a purpose in their work life. For instance, individuals with proactive schemas can get frustrated if they fail to have an active leading role (Carsten et al., 2010). Vanderslice (1988) also showed that individuals without formal power were motivated to lead certain tasks, providing further credence that downward following has some value for organizations.

Although downward following has obvious motivational and self-esteem benefits for those who lead upwards, it would not be desirable everywhere, particularly in political organizations where resources tend to always be contested (Mintzberg, 1985; Toegel and Jonsen, 2016). The idea of relinquishing influence towards individuals lower in a hierarchy may also conflict with the emphasis and glorification of leadership as currently taught in business schools (Alvesson and Sveningsson, 2003; Steffens and Haslam, 2022). Our claim is not that managers or supervisors should relinquish *all* their formal power or be *always* open to their employees’ influence (i.e., a level playing field); such situations could become chaotic and ultimately result in a lack of accountability and responsibility. What we instead encourage is an organizational context whereby influence can emerge from every member, irrespective of their role in the hierarchy. Organizations should signal to supervisors and managers that it is appropriate and even beneficial to follow their direct reports under the right circumstances.

We now offer a conceptual multilevel model to offer guidance for the study of the emergence of downward following behaviors.

## 4. A multilevel model of the emergence of downward following behaviors

**Figure 1:**
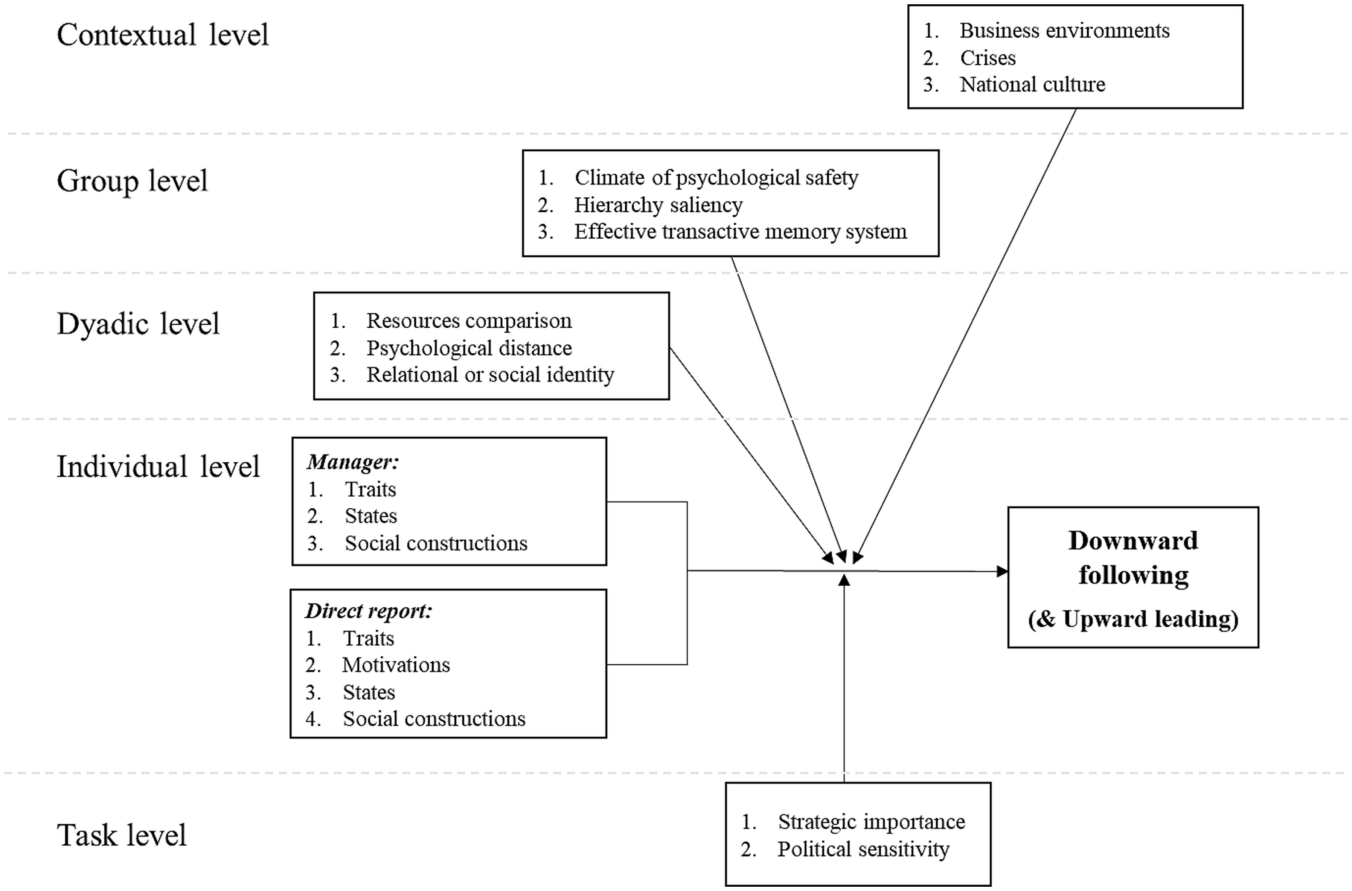
Multilevel conceptual model of the emergence of downward following (and upward leading).

Our proposed model (Figure 1) categorizes important themes for the study of the emergence of downward following. We offer antecedents at the individual level (both from a manager and direct report’s viewpoint), and boundary factors at the dyadic, task, group, and contextual levels. Seen as an organizing framework, our model provides future research directions and highlights where the field may focus its attention or broaden in the future. We borrow tentatively from neighboring literatures (e.g., voice, power, leader emergence) to build our model although we encourage the development of a unique downward following literature. Note also that this model does not aim to be exhaustive; it serves as a first step and will need to be tested, updated, and refined as new empirical knowledge will be gathered on the phenomenon.

### 4.1. Individual factors predicting downward following – manager/supervisor

To understand the emergence of downward following, we start by discussing managers’ traits and states, particularly their social constructions regarding individuals in positions of power. Managers high on the honesty-humility dimension (Lee and Ashton, 2004), and low on the dark triad (Paulhus and Williams, 2002) and social dominance (Pratto et al., 1994) are more likely to downward follow. Empirical evidence for instance suggests that humble CEOs are more likely to empower (Ou et al., 2014) and collaborate (Ou et al., 2018) with other individuals.

Because upward leading and downward following are associated with power relations, struggles, and tensions, it matters how managers construe and enact their formal role in an organization. Upward leading attempts may be rejected to protect one own’s turf (Mintzberg, 1985; Maner and Mead, 2010), particularly in climates where perceptions of power are salient (Galinsky et al., 2008). Thus, individuals construing power as a responsibility (rather than an opportunity; De Wit et al., 2017), having a personal sense of power (Sessions et al., 2020), and navigating stable power structures (Jordan et al., 2011; Feenstra et al., 2020; Gray et al., 2023) are less likely to see upward leading as a threat and are more likely to downward follow.

Furthermore, a social construction of employees as industrious individuals (Carsten et al., 2010; Whiteley et al., 2012) and a shared leadership structure schema (Derue and Ashford, 2010; Derue et al., 2015; Wellman et al., 2022) are two important characteristics that predict the emergence of downward following. Finally, we argue that managers who embrace to a certain degree a follower identity (Epitropaki et al., 2017) – even if only temporarily – will be more likely to downward follow because following is congruent with their identity.

### 4.2. Individual factors predicting downward following – employee/direct report

When predicting the emergence of downward following from an employee or direct report perspective, we rely on the literatures on informal leader emergence to inform our conceptual model (e.g., Judge et al., 2002; Hanna et al., 2021). We recognize that this discussion is not fundamentally new; however, it is new to consider employees’ characteristics as antecedents of downward following. We distinguish here between employees’ traits, motives, states, and social constructions.

Important employees’ traits predicting the emergence of downward following in their managers include general intelligence (Taggar et al., 1999; Judge et al., 2004), personality dimensions such as extraversion, conscientiousness, or emotional stability (Judge et al., 2002), proactivity (Grant and Ashford, 2008), and self-monitoring (Zaccaro et al., 1991). Motivational factors include employees’ motivation to lead (Chan and Drasgow, 2001; Luria and Berson, 2013), need for achievement and power (Mcclelland, 1975), and need for competence and growth (Ryan and Deci, 2000). Critical employees’ states predicting managers’ downward following refer to direct reports having high self-efficacy (Kwok et al., 2021), high leader identity (Miscenko et al., 2017), being legitimate to take a leading role (Aime et al., 2014), and being prototypical of a leader (Derue et al., 2015). Employees’ social constructions include low leadership risk perceptions (Zhang et al., 2020) and a proactive social construction of the employee role (Carsten et al., 2010), which are antecedents of managers emerging as downward followers.

All these employees’ characteristics increase the likelihood of a direct report’s upward leadership. As indicated earlier, a direct report’s leading attempt – which results from the different traits, motives, states, and social constructions listed above – is a necessary condition for the emergence of downward followership. For instance, Klasmeier et al. (2022) found that managerial empowering behaviors (a proxy of downward following behaviors) result from employees’ influential acts and behaviors, which suggests that managers are unlikely to emerge as downward followers if direct reports do not step up as upward leaders.

### 4.3. Dyadic factors moderating the emergence of downward following

We now discuss boundary conditions in the emergence of downward following, and more specifically the optimal conditions that favor its occurrence. Downward following is more likely to occur when employees have valuable resources to lead a specific task, situation, or project. For instance, direct reports who have expertise, skills, or knowledge to perform a specific task should lead this task (Aime et al., 2014). Also, when managers do not have sufficient time to appropriately lead a project, downward following is likely the best strategy. A low psychological distance, which may result from familiarity (Farh and Chen, 2018), interactions over time (Zhang et al., 2020), or similar status (Benson et al., 2016), may be necessary to build the required trust for downward followership to emerge. In conditions of high psychological distance, the situation is riskier for a manager because a direct report who leads may (intentionally or not) have misaligned goals. Also, a high psychological distance may prevent an accurate and timely collaboration between the manager and their direct report. Another dyadic characteristic pertains to the supervisor and direct reports sharing a relational or social identity (Sluss and Ashforth, 2007; Haslam et al., 2010). For instance, Oc et al. (2019) found that powerholders were more receptive to dissenting opinions and more open to ideas when they emerged from subordinates sharing a relational identity. Research on the social identity of leadership and followership (Platow et al., 2015; Steffens et al., 2021) suggests that a shared group or organizational identity is critical for downward following.

### 4.4. Task-related factors moderating the emergence of downward following

The kind of task or group project will also matter for the emergence of downward following. Managers should lead tasks and projects that have a strategic importance or may be subject to political behaviors such as rewards or promotion decisions (Ferris and Treadway, 2012). Similarly, a manager should lead tasks such as strategic work (e.g., translating the organizational strategy from the TMT at the micro level; Antonakis and House, 2014), goal setting (Ordóñez et al., 2009), or performance evaluations. In contrast, we expect more frequent downward following behaviors in situations such as implementing a group project requiring complex solutions, scanning the competitive environment, or performing creative tasks. Similarly, in the presence of long and large-scale projects, we expect the temporary emergence of downward following behaviors to be critical for the effectiveness of teams (Zhu et al., 2011). To summarize, downward followership will likely emerge for tasks or projects that are not too politically sensitive and require building on diverse information, expertise, or skills.

### 4.5. Group factors moderating the emergence of downward following

At the group level, we argue that downward following behaviors are more likely to emerge when the climate (i.e., group members’ shared perceptions of their work environment) is safe and allows for risk taking, when the hierarchy is not too salient, and when group members have an accurate transactive memory system. First, employees are more likely to influence managers in climates that are psychologically safe (Edmondson, 1999; Bradley et al., 2012), empowering (Seibert et al., 2004), or encouraging voice behaviors (Morrison et al., 2011). Such trustworthy climates foster risk taking behaviors, collaboration across group members, and shared decision making, which are all critically important for downward following. Second, downward followership and upward leadership will emerge when the hierarchy is not too salient, or at least when groups do not embrace an authority ranking model (Fiske, 1992). For instance, Klein et al. (2006) found that hierarchical environments prevent upward leading attempts; such context seems thus to preclude the emergence of downward following. Wellman (2017) suggests that formal hierarchical differentiation – when authority cues are salient – sends signals to group members that authority and formal power difference matters. We expect to observe a low likelihood of downward following in such environments. Third, group members (and managers in particular) need to have some appropriate meta-knowledge – in the form of an accurate transactive memory system - describing who is knowledgeable, expert, or skilled in a group (Lewis and Herndon, 2011). For instance, Xu et al. (2021) found that transactive memory systems were related to situationally-aligned leadership, which represents the emergence of leaders who fit the requirements of the situation. If managers cannot easily locate where (or whether) resources can be found in a team, we predict that managers will downward follow to a lesser extent.

### 4.6. Contextual factors moderating the emergence of downward following

Drawing on the distinction between omnibus and discrete contextual levels (Johns, 2006; Oc, 2018), we discuss how omnibus contextual factors moderate the emergence of downward following behaviors.^8^ We argue that slow changing business environments, low competitive industries, crises situations, and high-power distance as well as collectivist national cultures all represent factors limiting the emergence of downward following.

Certain business environmental conditions call for different sources of influence (i.e., leaders). For instance, fast changing, uncertain or very competitive business environments require adaptations, flexibility, and a high degree of collaboration to keep up with such environments (Brown and Eisenhardt, 1997). In contrast, slow changing, more stable, or monopolistic business environments offer clearer operational and performance standards that necessitate less downward following. Similarly, a crisis situation (at its onset) will limit the opportunity for downward following. During crisis periods, individuals look up to those in positions of power for certainty, direction, and a vision of the future (Bastardoz et al., 2022). Such tense situations do not call for downward followership, but rather formal leadership. Finally, managers or supervisors originating from national cultures high on the power distance or collectivism dimensions (Hofstede, 2011) are less likely to downward follow because such behaviors are not culturally expected; rather, managers are expected to “lead” and provide directions to the group (Blair and Bligh, 2018). Empirical evidence suggests that low power distance cultures relate positively to voice behaviors (Botero and Van Dyne, 2009) and empowering leadership (c.f., Sharma and Kirkman, 2015).

## 5. General discussion

As our systematic review uncovered, the study of followership suffers from three major issues that limit the validity of its scientific record. First, followers are inappropriately equated with direct reports. Second, the followership field does not study actual behaviors but mostly endogenous attitudes, cognitions, or perceptions of behaviors. Third, the study of downward following is non-existent in the literature although it may represent a critical source of organizational effectiveness. To counter these current limitations, we suggest taking a process approach to rethink what a follower and following behaviors actually mean. It also forces us to focus on actual following behaviors, which could be captured if studied at a micro-interaction level. We finally develop a conceptual model to encourage the study of downward following behaviors that aims at structuring and guiding future research of this critical phenomenon. We now discuss some implications of our review and proposed conceptual model.

Although we encourage the study of downward followership, we are not suggesting that it will or should be predominant in organizations. In fact, the majority of followership behaviors in organizations will likely emerge from direct reports or subordinates (Shamir and Eilam-Shamir, 2017). Our schemas and cognitive structures still imply that individuals in formal positions should take the lead, coordinate groups’ actions, or influence employees. Some managers may also consider that they have deserved their formal role by paying their dues, and that with this role comes the legitimacy and autonomy to direct and lead their direct reports. To change the schemas and scripts guiding manager-employee’s interactions, we need more role models of, and positive experiences with, downward following as well as individuals socialized in empowering and flatter organizations.

Even if unrealistic today, a change in the laymen perceptions of the terms followership and leadership would also be required to facilitate the acceptance of being a follower (Kniffin et al., 2020). Studying the emergence of downward following can help democratize the idea of being a follower and change the low reputation associated with following and followers (Hoption et al., 2012). To do so, we will need studies reporting on the positive outcomes associated with the emergence of downward following (e.g., group effectiveness, higher well-being, better organizational learning).

Downward following can become the source of competitive advantage for organizations if managers learn to do so effectively (e.g., when should they downward follow, or not?). As such, effective downward followership is an untapped resource that may become strategic for organizational success. Such development may make the training and development of followership skills more appealing. If, in our role as educators, we can approach (busy) managers and tell them schematically: “Here is a way to be more effective in your role as a manager, and it includes following person *x* under *y* or *z* circumstances while taking *a*, *b*, and *c* into account,” a followership identity may better resonate with them. Currently, followership development is not appealing because few managers want to develop the skills to be an effective direct report (as implied by a role-based approach) or know how, why, or when direct reports should have a more proactive approach to their role (Carsten, 2017; Hurwitz, 2017).

Organizations have a key role to play in the advent of downward following. Organizations need to nudge organizational actors’ preconception and cognitive theories towards more interactions across the hierarchy and responses to upward leading attempts. Organizations thus need to train, encourage, and reward their managers who downward follow. Even more importantly, they should provide space and opportunities for influence across hierarchical lines (Alvesson and Sveningsson, 2003), and this may take the role of a heterarchical culture (Aime et al., 2014). Organizations should also offer space to employees who want to upward lead, take initiative, and collaborate with their supervisors. It is critical that individuals with a proactive role schema (Carsten et al., 2010) have a working environment satisfying their needs.

Studying leading and following behaviors in micro-interaction episodes could help reduce concerns for under-represented groups who are affected by categorization and stereotyping. Because factors such as gender (Schein, 1973; Braun et al., 2017) or race (Rosette et al., 2008; McClean et al., 2018; Petsko and Rosette, 2023) still matter for the ideal implicit leadership and followership theories (ILTs and IFTs), studying naturally occurring behaviors using systematic coding schemes and trained coders may prevent the biases associated with categorizing and filling the gaps that generally results from questionnaire studies (Rush et al., 1977). Moving away from survey research may actually help reduce the stereotypical associations relating “followers” with under-represented groups.

Finally, our work is not without limitations. One limitation pertains to the simultaneous use of a systematic review and theory development within the same manuscript. Yet, an integrative review would have been more in line with our aim to redirect the field of followership (see Cronin and George, 2023). Another limitation refers to the absence, in our conceptual model, of outcomes associated with downward following behaviors. We treated downward following behaviors as a phenomenon to be explained. However, we expect downward following behaviors to be associated (and causally related) with a host of positive cognitive, affective, and behavioral outcomes for direct reports and managers. Future research will be needed to extend the nomological network of downward following behaviors. Our conceptual model is only a first step towards understanding and studying downward following; it will not only have to be extended, but also refined and trimmed as new empirical evidence becomes available.

## 6. Conclusion

Our systematic review indicates that the state of followership research is not where it should be. This manuscript aims at encouraging a fundamental rethink regarding what it actually means to be a follower. By introducing the concept of downward followership, we also aim to spark new research on an understudied, yet critical, phenomenon. This is sorely needed if the followership research aims to evolve with its time and remain consequential for researchers and practitioners alike.

## Author contributions

NB contributed to the conception of the manuscript and came up with the idea for the review and wrote the first draft of the manuscript. SA performed the review, analyzed the results, and contributed to manuscript revision. All authors contributed to the article and approved the submitted version.

## Footnotes

^1^For multi-studies article, we only coded for the first study unless the first study was a pilot study or when a later study included more followership constructs.

^2^Note that we used the terms “manager” and “supervisor” interchangeably in this manuscript.

^3^Retrieved on March 30, 2023.

^4^These direct reports’ behaviors may inform the followership literature if the behaviors help direct reports influence others to attain shared goals.

^5^Although, as Issues #1 and #2 indicate, such research has severe limitations and should not be labeled followership if there is no influence and shared goals.

^6^Although some constructs such as proactive behaviors, voice, or issue selling investigate the influential attempts of individuals in low formal power position, these constructs tend to be inherently change-oriented, do not center on shared goals, or focus on the leadership side.

^7^Cynics may reply that if reversing the lens on leadership by studying downward followership, should we not simply prescribe to abandon formal hierarchies (and thus study shared or collective leadership)? Although this suggestion may appear appealing, it seems quite unrealistic that formal authority and hierarchies will disappear from organizations anytime soon. We do not expect that self-managing teams or completely flat hierarchies (e.g., Zappo, Wikipedia) will soon become the norm, mainly because such organizational designs require a specific attention to the accountability and responsibility of individuals and groups.

^8^The discrete contextual factors are included in task-based, dyadic, and group factors moderating the emergence of downward following.
